# De novo mutations in *SCN1A* are associated with classic Rett syndrome: a case report

**DOI:** 10.1186/s12881-018-0700-z

**Published:** 2018-10-11

**Authors:** Mari Wold Henriksen, Kirstine Ravn, Benedicte Paus, Stephen von Tetzchner, Ola H Skjeldal

**Affiliations:** 1 Department of Neurology, Vestre Viken Hospital Trust, Drammen Hospital, P.O. Box 800, 3004 Drammen, Norway; 2 Institute of Clinical Medicine, Faculty of Medicine, University of Oslo, P.O. Box 1171, Blindern, 0318 Oslo, Norway; 3 Department of Clinical Genetics, Rigshospitalet, University of Copenhagen, Blegdamsvej 9, 2100 København Ø, Copenhagen, Denmark; 4 Department of Medical Genetics, Oslo University Hospital, P.O. Box 4950, 0424 Oslo, Norway; 5 Department of Psychology, University of Oslo, P.O. Box 1094, Blindern, 0317 Oslo, Norway; 6 Gillberg Neuropsychiatric Centre, Sahlgrenska University of Gothenburg, Kungsgatan 12, 41119 Gothenburg, Sweden

**Keywords:** Rett syndrome, Epilepsy, Genetics, *SCN1A*, Dravet syndrome

## Abstract

**Background:**

Rett syndrome (RTT) is a neurodevelopmental disorder. In more than 95% of females with classic RTT a pathogenic mutation in *MECP2* has been identified. This leaves a small fraction of classic cases with other genetic causes. So far, there has not been reported any other gene that may account for the majority of these cases.

**Case presentation:**

We describe two females who fulfill the diagnostic criteria for classic RTT, with pathogenic de novo mutations in *SCN1A*, which usually leads to Dravet syndrome. The developmental history and clinical features of these two females fits well with RTT, but they do have an unusual epileptic profile with early onset of seizures. Investigation of mRNA from one of the females showed a significantly reduced level of *MECP2* mRNA.

**Conclusions:**

To our knowledge, this is the first report suggesting that *SCN1A* mutations could account for a proportion of the females with classic RTT without *MECP2* mutations. As a consequence of these findings *SCN1A* should be considered in the molecular routine screening in *MECP2*-negative individuals with RTT and early onset epilepsy.

## Background

Rett syndrome (RTT, OMIM 312750) is a severe neurodevelopmental disorder, characterized by an apparently normal development the first 6–18 months, followed by regressive loss of acquired skills [1]. The current diagnostic criteria for classic RTT require a period of regression, loss of acquired purposeful hand skills and acquired spoken language (if any), gait abnormalities and stereotypic hand movements. Exclusion criteria include grossly abnormal psychomotor development in the first 6 months of life or known brain injury [1]. In more than 95% of females with classic and 50% with atypical RTT, a pathogenic mutation in *MECP2* has been identified [1]. Mutations in 69 other genes have in recent years been associated with RTT and RTT-like disorders [2, 3], including a girl with a RTT-like condition and a mutation in *SCN1A* [4]. The present study reports two females fulfilling the diagnostic criteria for classic RTT [1] with de novo mutations in *SCN1A*. Pathogenic mutations in *SCN1A* are known to cause Dravet syndrome [5] and have not to our knowledge been associated with classic Rett syndrome.

## Case presentations

### Case 1

**Fig. 1:**
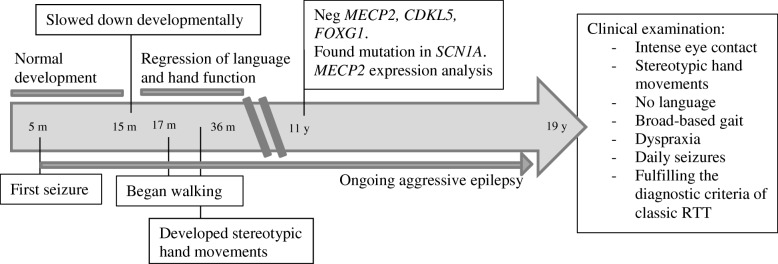
Timeline case 1

Case 1 is a 19 years old woman (for timeline see Fig. 1). She was born at 37 weeks gestation with a birth weight of 2890 g, length 47 cm, and a head circumference of 32 cm. Pre- and neonatal periods were normal. She had her first seizure, a prolonged febrile seizure, at 5 months of age. She developed afebrile focal seizures and intractable generalized seizures, both myotonic, tonic and tonic-clonic. She has had several episodes with convulsive status epilepticus. Her early development, however, was unremarkable. She developed normal hand function, including a pincer grip, and started to use a few words, 15 at the most. She began walking independently at 17 months. However, from around 15 months of age her development slowed down and she gradually lost acquired skills. She stopped using her hands, her words disappeared and her gait became broad-based and ataxic. She developed midline rubbing hand stereotypies, although not very intense, and bruxism. She often had breath-holding spells and infrequently she hyperventilated. Her sleep pattern was impaired with night time screaming spells and occasionally laughing spells. Between one and 2 years of age, she developed autistic traits. She had a deceleration of head growth from 50th to 10th percentile.

The clinical examination at 19 years revealed a woman with intense eye contact and ongoing stereotypic hand movements with hand dyspraxia. She had a broad-based gait with notable ataxia. Breath holding and teeth grinding were observed. She was only 141 cm tall, but had normal weight for height. Her musculature was generally hypotonic and she had a slight scoliosis. Her epilepsy was still aggressive with daily seizures (focal, tonic and tonic-clonic), despite intense anti-epileptic treatment. Her clinical signs and symptoms were consistent with classic RTT, fulfilling the criteria of this disorder.

**Fig. 2:**
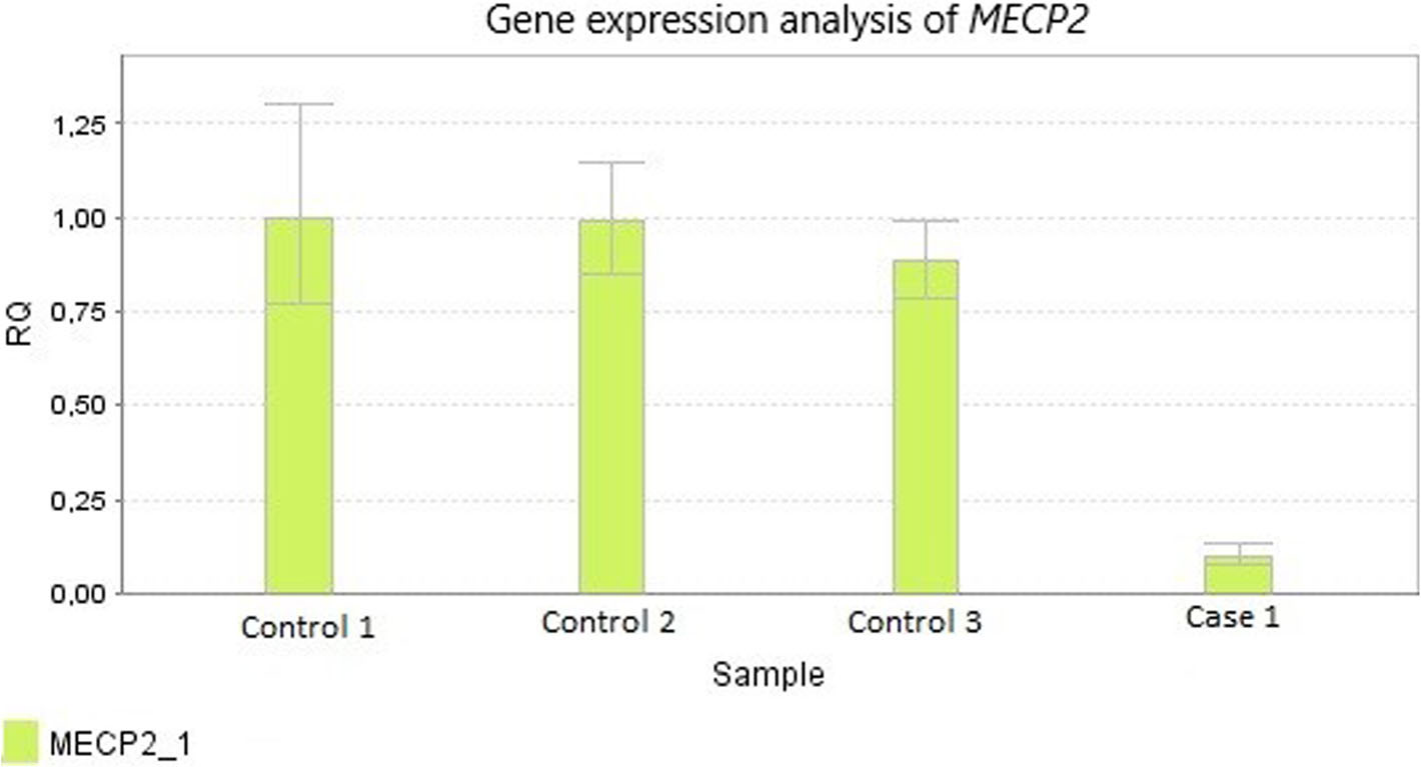
The relative expression level of *MECP2* (the alternative transcripts coding for isoforms MeCP2_E1) in cultured fibroblasts of three female controls and Case 1. The *MECP2* gene expression analysis was performed on mRNA isolated from her fibroblasts, using Applied Biosystems 7500 Fast Real-Time PCR System (ABI, Foster City, CA), with pre-designed assay (TaqMan MGB probe HS01598237). *GAPDH* served as an endogenous control. The RQmin and RQmax with the confidence interval set at 95%:Female1; RQ Min 0.77, RQ Max 1.29, Female2; RQ Min 0.85, RQ Max 1.14, Female3; RQ Min 0.787, RQ Max 0,99, Patient; RQ Min 0.07, RQ Max 0,13

CT and MRI scans of the brain were unremarkable. At the age of eleven *MECP2*, *CDKL5*, and *FOXG1* were analyzed with Sanger sequencing of all exons with flanking intron regions and MLPA kits P015C, P395 and P189 from MRC-Holland, all with normal results. Due to the aggressive epilepsy *SCN1A* was Sanger sequenced and this disclosed the novel splice variant NG_011906.1:g.76169G > C, (NM_001165963.2): c.4284 + 1G > C. Using Alamut Visual software (Interactive Biosoftware, France) and the guidelines of American College of Medical Genetics and Genomics and the association for Molecular Pathology (ACMG) [6], this variant was scored as pathogenic. Parental testing indicated that the mutation was de novo. Two splice mutations (c.4284 + 1G > T and c.4284 + 1G > A) affecting the same splice site, have previously been reported in Dravet syndrome [7, 8]. Because she fulfilled the criteria for RTT, but no mutation in *MECP2* was identified, a *MECP2* gene expression analysis, performed on mRNA isolated from her fibroblasts was performed. This analysis indicated that her *MECP2* expression level was more than 80% reduced compared to three female controls (Fig. 2).

### Case 2

**Fig. 3:**
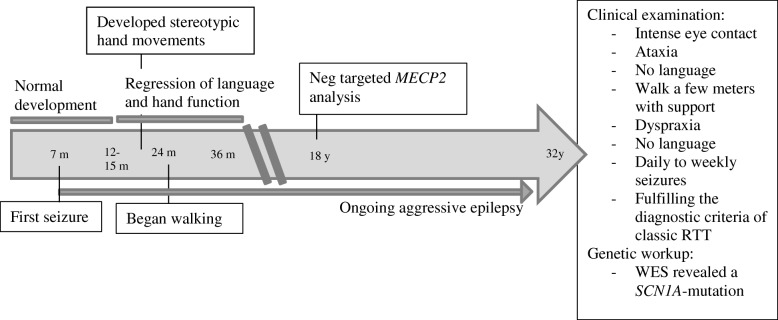
Timeline case 2

Case 2 is a 32 years old woman (for timeline see Fig. 3). She was born at 40 weeks of gestation with a birth weight of 3830 g, length 52 cm, and a head circumference of 36 cm. Pre- and neonatal periods were normal. At 7 months, she had her first seizure, a febrile bilateral tonic-clonic seizure. Between one and 2 years of age her epilepsy became more severe, with daily generalized seizures. The frequency of seizures declined somewhat when she reached school age, but her epilepsy remained drug resistant, with several bilateral tonic-clonic seizures every week. Besides the epilepsy, her development was apparently normal the first 12–15 months. She sat independently at 7 months. At 1 year, she used a few words and had an appropriate use of hands. She learned to walk when she was 24 months old. When she was between 12 to 15 months of age she started to lose acquired skills. Her hand function deteriorated gradually, her words disappeared and she no longer seemed to show interest in her surroundings. She developed bruxism and hand-washing stereotypies. She could walk independently until school age, but then she gradually needed support when walking. Through childhood her sleep pattern was significantly disturbed with both screaming and laughing spells. Her respiration has however never been affected.

The clinical examination revealed a 32 years old woman who could walk a few meters with support, had ataxic and apraxic hand movements, but not hand stereotypies. She had no language but gave intense eye contact. Her muscle tone was normal. She had a slight scoliosis. Her epilepsy was still a major concern, with daily to weekly bilateral tonic-clonic seizures. She fulfilled the criteria of classic RTT.

Genetic analyses of *MECP2* at the age of 18 gave negative results (Sanger sequencing and MLPA kit P015 from MRC-Holland). As a participant in a national survey of females with RTT she was recently retested by applying whole exome sequencing (WES) using Agilent SureSelect Target Enrichment Kit (Agilent Technologies, Santa Clara, CA) on Illumina HiSeq 2500 with pair-end runs. Alignment, mapping, and variant calling were performed using Genome Analysis Tool Kit (GATK). Reads were mapped to the reference sequence (GRCh37/hg19). Following bioinformatic filtration, analysis of coding regions and intron/exon boundaries of 1479 predefined genes (including *FOXG1*, *CDKL5* and *SCN1A* with a 100% coverage at a depth > 10×) was performed. WES disclosed the variant, NG_011906.1:g.76130G > T, NM_001165963.1: c.4246 G > T, p.(Asp1416Tyr) in *SCN1A.* Using Alamut Visual software (Interactive Biosoftware, France) and ACMG criteria [6] this novel variant was scored as pathogenic. Parental testing indicated that the mutation was de novo. This is a **novel** variant, but mutations affecting the same amino acid have been reported in Dravet syndrome [9].

## Discussion and conclusions

We present two females with clinical pictures consistent with classic RTT and who fulfill the diagnostic criteria for this disorder [1], but without mutations in the coding regions of *MECP2, CDKL5* and *FOXG1*. However, deep intronic mutations and duplications/deletions of exons not covered by the MLPA analysis, have not been excluded.

Further genetic analyses revealed presumed pathogenic de novo mutations in *SCN1A* in both. More than 80% of individuals with pathogenic mutations in *SCN1A* have Dravet syndrome [10]. Both females do have clinical features associated with this syndrome, like early seizure onset, prolonged febrile seizures, status epilepticus, and drug resistant epilepsy [5]. Dravet syndrome has no clearly defined diagnostic criteria and the phenotypic spectrum is wide. These case reports show that there may be a clinical overlap between features of RTT and other neurodevelopmental disorders, such as Dravet syndrome. This is a challenge for disease classification and diagnosis. Strict and robust criteria are necessary for making consistent diagnoses and sorting out differential diagnosis. Recognizing potential confusion, the revised RTT criteria suggest specifying both phenotype and mutation [1].

Finding the molecular basis is important in clinical practice, for prognosis and genetic counseling, and it may have implications for treatment. It may also be essential for better understanding of the pathophysiology. For instance, in Case 1, harboring the *SCN1A* splice site mutation, quantitative gene expression analyses showed a reduced level of *MECP2* mRNA in fibroblasts, although no *MECP2* mutation was detected. In order to evaluate the significance of this finding further research is demanded. Both females presented here participated in a national survey of the Norwegian population of females with RTT. This survey includes 93 participants with RTT and RTT-like disorders, 74 with classic RTT. A total of 12 participants did not have mutations in *MECP2,* three in the group with classic RTT, including the two females presented here (2.7% of the participants with classic RTT in this cohort). The presence of these two cases in the Norwegian RTT cohort indicates that *SCN1A* mutations could account for a significant part of the population of females with classic RTT without *MECP2* mutations. Although fulfilling the diagnostic criteria for classic RTT their epileptic profile is atypical with early seizure onset and prolonged febrile seizures. The possibility that the two females’ phenotype might be a result of two mutations, one *SCN1A* and one rare intronic variation in *MECP2* or *CDKL5,* seems unlikely with our present knowledge.

In the cohort of 74 individuals with classic RTT these two individuals and two others were the only ones with seizure onset before regression. The findings in this paper could lead to justifying the inclusion of *SCN1A* in the molecular routine screening for *MECP2*-negative individuals with RTT and early onset epilepsy.

## Abbreviations

ACMG guidelines: Guidelines of American College of Medical Genetics and Genomics and the association for Molecular Pathology
GATK: Genome Analysis Tool Kit
MLPA: Multiplex Ligation-dependent Probe Amplification
RTT: Rett syndrome
WES: Whole Exom Sequencing
